# Midlife Periodontal Disease and Cognitive Functioning: Evidence from High School and Beyond

**DOI:** 10.1002/alz.088355

**Published:** 2025-01-09

**Authors:** John Robert Warren, Ryan Demmer, Eric Grodsky, Chandra Muller

**Affiliations:** ^1^ University of Minnesota, Minneapolis, MN USA; ^2^ Mayo Clinic, Rochester, MN USA; ^3^ University of Wisconsin ‐ Madison, Madison, WI USA; ^4^ University of Texas at Austin, Austin, TX USA

## Abstract

**Background:**

Periodontal disease is a prevalent, preventable, and highly treatable oral infection that is (1) stratified by sociodemographic, educational, and spatial factors and (2) is believed to impact cognitive impairment and ADRD risk. However, there is very little information from community‐based, population‐representative, longitudinal studies about the association between periodontal disease and midlife cognitive functioning net of early life confounders.

**Method:**

We use data from the U.S. High School and Beyond (HSB) cohort study, which has followed a nationally representative sample of ∼25,500 people from high school in 1980 through midlife in 2021‐22. HSB data from the 1980s includes rich information about educational contexts, opportunities, and outcomes; early life socioeconomic and family circumstances; spatial location; and demographic group memberships. HSB data from 2021‐22 include indicators of periodontal disease diagnosis and multiple measures of cognitive functioning.

**Result:**

People who have been diagnosed with periodontal disease score worse on measures of memory, executive function, and other domains of cognitive functioning at midlife. These associations are only partly attributable to early life confounders. Some ─ but not all ─ of the association can be attributed to the mediating role of diabetes and CVD.

**Conclusion:**

Periodontal disease is a preventable and treatable risk factor for subsequent cognitive impairment. Because risk of periodontal disease is stratified by demographic, educational, and other social factors, periodontal disease may also be important in shaping sociodemographic gradients in midlife cognitive functioning. This is the first population‐representative study using data from a community‐based cohort that has been followed from adolescence through midlife. As such, this work provides important evidence about the role of periodontal disease in stratifying cognitive outcomes.

